# Incidence and Correlates of HIV-1 RNA Detection in the Breast Milk of Women Receiving HAART for the Prevention of HIV-1 Transmission

**DOI:** 10.1371/journal.pone.0029777

**Published:** 2012-01-11

**Authors:** Jennifer A. Slyker, Michael H. Chung, Dara A. Lehman, James Kiarie, John Kinuthia, Sarah Holte, Kenneth Tapia, Francis Njiri, Julie Overbaugh, Grace John-Stewart

**Affiliations:** 1 Department of Global Health, University of Washington, Seattle, Washington, United States of America; 2 Department of Medicine, University of Washington, Seattle, Washington, United States of America; 3 Department of Obstetrics and Gynaecology, Kenyatta National Hospital, Nairobi, Kenya; 4 Division of Human Biology, Fred Hutchinson Cancer Research Center, Seattle, Washington, United States of America; 5 Department of Obstetrics and Gynaecology, University of Nairobi, Nairobi, Kenya; 6 Department of Biostatistics, University of Washington, Seattle, Washington, United States of America; 7 Department of Epidemiology, University of Washington, Seattle, Washington, United States of America; University of Cape Town, South Africa

## Abstract

**Background:**

The incidence and correlates of breast milk HIV-1 RNA detection were determined in intensively sampled women receiving highly active antiretroviral therapy (HAART) for the prevention of mother-to-child HIV-1 transmission.

**Methods:**

Women initiated HAART at 34 weeks of pregnancy. Breast milk was collected every 2–5 days during 1 month postpartum for measurements of cell-associated HIV DNA and cell-free HIV RNA. Plasma and breast milk were also collected at 2 weeks, 1, 3 and 6 months for concurrent HIV-1 RNA and DNA measurements. Regression was used to identify cofactors for breast milk HIV-1 RNA detection.

**Results:**

Of 259 breast milk specimens from 25 women receiving HAART, 34 had detectable HIV-1 RNA (13%, incidence 1.4 episodes/100 person-days 95% CI = 0.97–1.9). Fourteen of 25 (56%) women had detectable breast milk HIV-1 RNA [mean 2.5 log_10_ copies/ml (range 2.0–3.9)] at least once. HIV-1 DNA was consistently detected in breast milk cells despite HAART, and increased slowly over time, at a rate of approximately 1 copy/10^6^ cells per day (p = 0.02). Baseline CD4, plasma viral load, HAART duration, and frequency of breast problems were similar in women with and without detectable breast milk HIV-1 RNA. Women with detectable breast milk HIV-1 RNA were more likely to be primiparous than women without (36% vs 0%, p = 0.05). Plasma HIV-1 RNA detection (OR = 9.0, 95%CI = 1.8–44) and plasma HIV-1 RNA levels (OR = 12, 95% CI = 2.5–56) were strongly associated with concurrent detection of breast milk HIV-1 RNA. However, no association was found between breast milk HIV-1 DNA level and concurrent breast milk HIV-1 RNA detection (OR = 0.96, 95%CI = 0.54–1.7).

**Conclusions:**

The majority of women on HAART had episodic detection of breast milk HIV-1 RNA. Breast milk HIV-1 RNA detection was associated with systemic viral burden rather than breast milk HIV-1 DNA.

## Introduction

Both HIV-1 cell-free RNA and cell-associated DNA are associated with the risk of breast milk transmission [1], [2], [3]. In the absence of antiretroviral therapy, HIV-1 RNA levels in the breast milk are typically 1–2 log lower than in blood, and a proportion of women have no detectable breast milk HIV-1 RNA [1], [2], [4], [5]. HAART initiated during pregnancy significantly decreases breast milk HIV-1 RNA levels and decreases transmission risk [6], [7]. However, antiretroviral therapy has distinct effects on cell-free and cell-associated virus in the breast milk. In two separate clinical trials, women on HAART had significantly decreased breast milk HIV-1 RNA but not DNA [4], [6]. It has been hypothesized that HAART results in the rapid clearance of HIV-infected, activated CD4+ T cells, but has a smaller effect on the reservoir of virus in resting monocytes; hence the clearance of infected CD4+ T cells during HAART could affect cell-free RNA loads, but have an insignificant effect on overall HIV-1 cell-associated DNA levels [4].

Episodic low-level plasma RNA viremia is not uncommon in adults with stable viral suppression on HAART [8], [9], [10], [11]. Episodic detection of plasma HIV-1 RNA may result from fluctuations in adherence, drops in drug levels, emergence of resistant virus, transient increases in immune activation, or activation of latently infected cells [12], [13]. Episodic detection of breast milk HIV-1 RNA has been described in antiretroviral naïve women and women receiving single-dose nevirapine [1], [14] but to our knowledge, has not been studied in women receiving HAART. Since HAART for PMTCT does not offer complete protection from transmission during breastfeeding [15], [16], [17], it is plausible that incomplete viral suppression in breast milk may have significance for transmission at a population level. As rapidly evolving guidelines and expanding programs will increase numbers of women receiving HAART during breastfeeding, defining determinants of breast milk HIV-1 breakthrough during HAART may contribute to further strategic improvements in PMTCT interventions. In this study among women with collection of breast milk at frequent intervals (every 2–5 days) we determined the incidence, magnitude, and correlates of breast milk HIV-1 RNA detection among women starting HAART during pregnancy for PMTCT, and developed longitudinal regression models exploring the relationship between viral replication in different biological compartments.

## Methods

### Study participants and follow-up

This is a retrospective study utilizing cryopreserved specimens and repository data from a previously conducted randomized clinical trial (NCT00167674). The trial was approved by the Institutional Review Board (IRB) at the University of Washington (Seattle, WA, USA) and the Ethics in Research Committee (ERC) at Kenyatta National Hospital (Nairobi, Kenya) and all clinical investigations were conducted according to the principles expressed in the Declaration of Helsinki. All study participants provided written, informed consent for study participation on behalf of themselves and their infants. The primary data analysis, methods of recruitment, randomization, and follow-up have been described elsewhere [4], [7]. In brief, pregnant women were recruited from the Mathare North City Council Clinic in Nairobi, Kenya and were invited to enroll if they were HIV-1 seropositive, had hemoglobin ≥8 g/dl, had no previous exposure to antiretroviral medications, were ≥18 years of age, agreed to home visits, and resided in the clinic catchment area. Women with CD4 counts ≥200 and ≤500 cells/mm^3^ (who were not HAART-eligible according to contemporaneous guidelines) were randomized at approximately 34 weeks gestation to HAART or short-course antenatal zidovudine (ZDV) plus single-dose nevirapine (NVP) at delivery. This current report is restricted to the subset of 25 women in the HAART arm; breast milk viral rebound in women from the ZDV/NVP arm will be discussed in a separate report (S. Holte, in preparation). Women in the HAART arm received zidovudine, lamivudine and nevirapine from 34 weeks gestation until 6 months after delivery.

Throughout follow-up, infants were screened for HIV-1 DNA from dried blood spots as previously described [18]. Maternal plasma was collected at 32 weeks gestation, delivery, 2 weeks postpartum, then 1, 3 and 6 months after delivery. Breast milk was collected 1 to 3 times a week over the first month postpartum by peer counselors who visited the home and observed mothers manually express milk from a single breast into a sterile container. After the first month, breast milk was collected at the third and sixth months postpartum. Between 5 and 40 ml of breast milk was collected at each time point. CD4 counts were measured from blood specimens using flow cytometry. HIV-1 plasma viral loads were measured using the Gen-Probe assay (Gen-Probe Inc., San Diego, CA, USA) as previously described [19], the limit of detection for plasma samples was 200 copies/ml.

At each clinic visit (months 1, 3 and 6) the number of pills dispensed was recorded and women brought in unused pills for counting. Adherence was estimated for each drug as the percentage of pills taken over each study visit interval, assuming the women had taken their morning dose on clinic day (unless reporting otherwise):
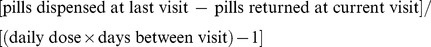



### Measurement of breast milk HIV-1 RNA and DNA

The processing of breast milk samples in this study have been described elsewhere [4]. In brief, breast milk samples were centrifuged at 710×g for 20 minutes and the lipid layer was discarded. The supernatant was frozen at −70°C. The cell pellet was resuspended in freezing media (70% RPMI , 20% fetal calf serum, and 10% DMSO) and stored in liquid nitrogen.

HIV-1 RNA was measured from 100 µl of breast milk supernatant using the GenProbe assay, the lower limit of detection for the breast milk specimens was 100 copies/ml.

Cell-associated DNA was extracted from 500 µl of breast milk cell suspension using the QIAmp DNA mini kit (Qiagen, Valencia, California) and HIV-1 pol and β-actin DNA were quantified using real-time PCR as previously described [1], [20]. The lower limit of detection was 1 copy/reaction, HIV-1 DNA levels were normalized to the number of cells tested (number of β-actin copies).

### Statistical analysis

StataSE version 11.1 (College Station, Texas) was used for all analyses. Viral load measurements below the limit of detection were recoded to the mid-point between zero and the limit of detection for each assay. The Mann-Whitney U test was used to make non-parametric comparisons between variables and the independent t test was used for comparison of means. The incidence of viral detection was calculated using Cox regression to estimate the recurrent failure rate; data were censored at the end of HAART or 180 days postpartum, whichever came first. Generalized estimating equations (GEE) were used to estimate changes in HIV-1 DNA load over time, and to examine associations between breast milk and plasma loads in longitudinal models. Models for continuous outcomes used the identity link and Gaussian errors; models for dichotomous outcomes used the logistic link and binomial errors. All regression models were constructed using exchangeable correlation matrix and robust standard errors. Because many assays were below the limit of detection, breast milk HIV-1 RNA was modeled as a dichotomized outcome (detected/not detected). Plasma RNA was included as alternately a dichotomous (detected/not detected) or continuous (viral load) covariate. Since few specimens had undetectable HIV-1 DNA, this was included only as a continuous variable. Because there were many unmatched breast milk cell – supernatant pairs following month 1, modeling for breast milk DNA are restricted to the first month postpartum. All p-values are two-tailed.

## Results

### Study population

Between November 2003–March 2005, 58 women were enrolled in the randomized trial, of whom 30 were randomized to receive HAART; 2 were lost prior to delivery, 2 experienced stillbirths, and 1 discontinued HAART before delivery. The current study focuses on the 25 women who continued taking HAART through delivery and 6 months of lactation. At delivery, median duration of HAART in the cohort was 36 days (IQR = 25–48).

A total of 353 breast milk HIV-1 RNA assays were conducted, 259 of which were obtained during HAART. In women who were still lactating, plasma and breast milk specimens were also collected after HAART (94 specimens) interruption for studies of viral rebound (Holte, in preparation), and are not analyzed here. Breast milk HIV-1 DNA was additionally measured longitudinally during the first 3 months of follow-up in the subset of 17 women that had breast milk cell samples available (168 measurements) [4].

### Breast milk HIV-1 RNA detection and suppression in women receiving HAART for PMTCT

**Figure 1:**
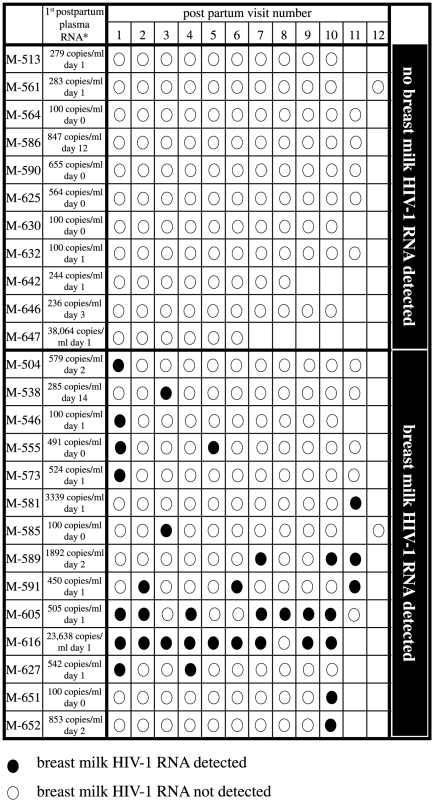
Detection of breast milk HIV-1 RNA in women receiving HAART for PMTCT. The detection of HIV-1 RNA is shown for 25 women on HAART postpartum. Visits were ranked by date, and are shown only for visits while HAART was being administered. *The first postpartum HIV-1 plasma RNA measurement is provided for each woman. M-586 and -538 did not have a plasma viral load measured within a week of delivery; plasma viral loads shown for these two women coincide with visit 1.

Plasma HIV-1 RNA viral load declined rapidly after women started HAART (Figure S1), but because of the relatively late start of HAART, the majority of women in this study had detectable plasma HIV-1 RNA at their first postpartum visit (19/25, 76%). Of 259 breast milk specimens assessed during HAART, 34 had detectable HIV-1 RNA (13%); the mean level of detectable HIV-1 RNA was 2.5 log_10_ copies/ml (±SD 0.58), and ranged from 2.0 to 3.9 log_10_ copies/ml. A total of 14 women (56%) had detection of breast milk HIV-1 RNA at least once, and 11 women (44%) never had detectable breast milk HIV-1 RNA (Figure 1). The majority of women with detectable breast milk HIV-1 RNA had episodic detection of virus (12 women, virus detected at less than half their visits). Two women had detection of HIV-1 RNA at more than half of their visits (M-605 and M-616).

The 25 women provided a total of 2509 person-days of follow-up during the first 9 months postpartum. The incidence of breast milk HIV-1 RNA detection overall was 1.4 episodes/100 person-days [95%CI 0.97–1.9]. Among women with detectable HIV-1 RNA (1342 person-days of follow-up), the incidence of detectable breast milk HIV-1 RNA was 2.5 episodes/100 person-days [95%CI 1.8–3.5].

### Suppression of breast milk HIV-1 RNA despite an active breast milk cellular reservoir

**Figure 2:**
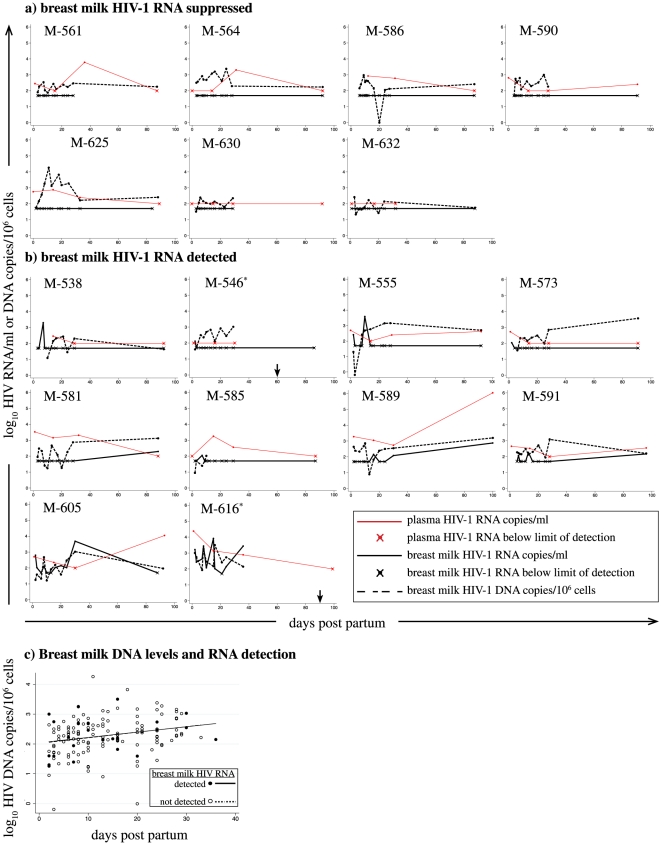
Breast milk HIV-1 RNA, cell-associated DNA, and plasma RNA viral loads during HAART for PMTCT. Breast milk HIV-1 RNA (solid line), breast milk HIV-1 DNA (dashed line), and plasma RNA (red line) are shown for women with a) suppression of HIV-1 RNA in breast milk and b) detection of HIV-1 RNA in breast milk. All women with cell-associated virus measured are shown. *M-546 and -616 stopped HAART before 100 days; arrows show last date HAART dispensed for these two women. Data points marked with an “x” indicate the measurement taken was at or below the assay limit of detection. c) Scatter plot shows levels of breast milk HIV-1 DNA at breast milk HIV-1 RNA detectable (solid circles and solid line) and undetectable (open circles and dashed line) time-points during HAART. Linear models are fit to the data. Data shown are limited to the first month postpartum, because the majority of HIV-1 DNA measurements (154/163) were collected during this time interval.

Seventeen of 25 women had detectable cell associated HIV-1 DNA at one or more visits. Breast milk HIV-1 DNA was detected at high levels despite HAART, and was found in both women with undetectable (Figure 2a) and detectable (Figure 2b) breast milk HIV-1 RNA. Generalized estimating equations (GEE) were used to compare the level of breast milk HIV-1 DNA measured at HIV-1 RNA detectable and undetectable time-points (Figure 2c). Overall, the level of breast milk HIV-1 DNA increased over time, as previously described [4], at a rate of ∼1 copy/10^6^ cells per day [slope = 1.041, 95%CI = 1.0073–1.076, p = 0.02]. We observed no difference in breast milk HIV-1 DNA load measured at HIV-1 RNA detectable and undetectable time-points; both the rate of increase (p = 0.99) and level (p = 0.98) of HIV-1 DNA were similar.

### Comparison of women with detectable and undetectable breast milk cell-free HIV-1 RNA detection

Obstetric, immunologic, and virologic characteristics were compared between women with detectable and undetectable breast milk HIV-1 RNA (Table 1). Women in the two groups were similar in their duration of HAART at delivery, pre-HAART disease status, gestational age at HAART initiation, delivery plasma viral loads and CD4 counts, and adherence. Availability of the participant, ability to express breast milk at discrete visits, and confidentiality concerns during home visits resulted in some women having more breast milk specimens collected than others. There was no difference in the follow-up time or the number of visits assessed between the two groups, and they also breastfed for a similar duration of time. Women with detectable breast milk HIV-1 RNA were more likely to be first-time mothers than women with undetectable breast milk HIV-1 RNA; 36% (5/14) of women with detectable breast milk HIV-1 RNA and none (0/11) of the women with undetectable breast milk HIV-1 RNA were primiparous (p = 0.05). There was a trend for women with detectable breast milk HIV-1 RNA to be younger than women with undetectable breast milk HIV-1 RNA (26, IQR 25–30 versus 29 years, IQR 22–28; p = 0.08, respectively). As expected, primiparous women were also younger in age (data not shown), suggesting age confounds the relationship between primiparity and detection of breast milk HIV-1 RNA.

**Table 1:** Comparison of women by detection of breast milk HIV-1 RNA.

	Breast milk HIV-1 RNA		
	Never detected	Everdetected	P
	(N = 11)	(N = 14)	
*Sampling and follow-up*			
Number of follow-up visits	11 (10–11)	11 (10–11)	0.4
Days on HAART at delivery	35 (26–53)	37 (25–42)	0.8
Days of postpartum follow-up on HAART	92 (89–122)	92 (88–93)	0.5
*Obstetric*			
Age (years)	29 (25–30)	26 (22–28)	0.08
Gravidity	2 (1–2)	1 (0–3)	0.4
Parity	1 (1–2)	1 (0–2)	0.3
Primiparous	0% (0/11)	36% (5/14)	0.05
*Breastfeeding and breast problems*			
Duration of breastfeeding in days	182 (155–184)	182 (153–186)	0.8
Clinical mastitis*	18% (2/11)	7.1% (1/14)	0.6
Cracked nipples*	36% (4/11)	29% (4/14)	1.0
Any breast problem**	45% (5/11)	50% (7/14)	1.0
*Baseline HIV disease pre-HAART*			
HIV-1 log_10_ RNA copies/ml plasma VL	4.8 (4.6–4.9)	4.9 (4.7–5.0)	0.7
CD4 count (cells/mm^3^)	279 (257–351)	280 (248–421)	0.8
*HAART administration and adherence*			
Days on HAART at delivery	35 (26–53)	37 (25–42)	0.8
Combivir adherence (% pills taken)	96% (94–97)	96% (95–99)	0.9
Nevirapine adherence (% pills taken)	97% (95–99)	97% (95–100)	0.7
Total adherence (% pills taken)	97% (96–98)	96% (95–100)	1.0
*Immunologic & virologic status at delivery* ***			
CD4 count (cells/mm^3^)	445 (412–591)	385 (287–497)	0.3
HIV-1 log_10_ RNA copies/ml plasma VL	2.4 (2.0–2.8)	2.7 (2.5–2.9)	0.3
Plasma RNA detectable	73% (8/11)	79% (11/14)	0.5

Notes. Median (IQR) or frequency (n/N) are shown for each correlate. VL, viral load; p-d, person-days 95%CI, 95% confidence interval;

*At any time (includes antenatal and on HAART).

**Includes clinical mastitis, cracked nipples, localized swelling, and breast abscess.

***Measurements at delivery or first postpartum visit.

We collected detailed clinical data (self-report and clinical examination) during follow-up. However, women were generally well at enrollment, and we did not have adequate cases to detect associations between intercurrent illness and detection of RNA in milk/plasma (data not shown). There was 1 case of smear-positive malaria, and this woman had detectable breast milk RNA, and undetectable plasma RNA at the visit. Breast infections or breast problems were not increased in women with detectable breast milk HIV-1 RNA; three cases of clinical mastitis were identified and only one of these had detectable breast milk HIV-1 RNA (p = 0.6). Overall, the frequency of clinically apparent breast problems was similar between women with detectable (50%, 7/14) and undetectable (45%, 5/11; p = 1.0) breast milk HIV-1 RNA.

### Breast milk HIV-1 RNA detection is strongly associated with concurrent plasma RNA but not breast milk DNA

Generalized estimating equations (GEE) were used to determine the association between systemic (plasma RNA) and local (breast milk cell-associated DNA) HIV-1 replication and breast milk HIV-1 RNA (Table 2) during HAART. Detection of HIV-1 RNA in plasma was associated with a 9-fold increased odds [95% CI = 1.8–44] of detecting HIV-1 RNA in the concurrently collected breast milk specimen. The level of plasma RNA was also strongly correlated with the detection of breast milk HIV-1 RNA (OR = 12, 95%CI = 2.5–56). Duration on HAART was not significantly associated with breast milk HIV-1 RNA detection (p = 0.5).

**Table 2:** Predictors of concurrent HIV-1 cell-free RNA detection in breast milk during PMTCT HAART.

	N	Unadjusted OR [95% CI]	Adjusted for time on HAART aOR [95% CI]
*All assays*			
Days on HAART	25	1.0 [0.99–1.0], p = 0.5	
Plasma HIV-1 RNA level	24	12 [2.5–56], p = 0.002	13 [2.0–92], p = 0.008
Plasma HIV-1 RNA detected	24	9.0 [1.8–44], p = 0.007	9.0 [1.8–44], p = 0.007
Breast milk HIV-1 DNA level*	17	0.96 [0.54–1.7], p = 0.9	1.0 [0.56–1.8], p = 1.0
*Assays <250 copies/ml breast milk HIV-1 RNA* **			
Days on HAART	25	1.0 [0.98–1.0], p = 1.0	
Plasma HIV-1 RNA level	22	14 [3.1–59], p<0.001	18 [2.7–124], p = 0.003
Plasma HIV-1 RNA detected	22	12 [1.3–115], p = 0.03	12 [1.3–114], p = 0.03
Breast milk HIV-1 DNA level*	17	1.0 [0.48–2.1], p = 1.0	1.0 [0.48–2.2], p = 0.9

Notes. N, number of subjects in analysis. The association between breast milk HIV-1 RNA detection and each covariate is examined separately in an unadjusted model, and adjusted for days on HAART.

*Analysis for breast milk HIV-1 DNA was restricted to the first month postpartum because the majority of HIV-1 DNA measurements (154/163) were made during this time interval.

**In all models adjusted for time, days on HAART remained insignificant with an OR of approximately 1.0 (not shown).

Interestingly, we did not find any association between the level of breast milk HIV-1 DNA and breast milk HIV-1 RNA detection (OR = 0.96, 95%CI = 0.54–1.7). Because breast milk HIV-1 DNA levels increased and plasma RNA levels declined over time, we also adjusted for days on HAART. Adjusting for HAART duration did not change relationships observed in unadjusted models; no association was observed between breast milk HIV-1 DNA levels and breast milk HIV-1 RNA detection (adjusted OR (aOR) = 1.0, 95%CI = 0.56–1.8). Plasma RNA detection (aOR = 9.0, 95%CI = 1.8–44) and plasma RNA load (aOR = 13, 95%CI = 2.0–92) remained significant predictors of breast milk HIV-1 RNA detection.

Finally, we performed a sensitivity analysis to determine whether a few high level viral loads were driving the observed associations, excluding breast milk HIV-1 RNA loads of ≥250 copies/ml. There were only 5 breast milk HIV-1 RNA loads ≥250 copies/ml that had concurrent plasma RNA load measurements; exclusion of these 5 higher-level breast milk HIV-1 RNA load measurements did not change any of the relationships observed in either the univariate or multivariate models (Table 2), indicating the associations observed in these models were not the result of a small number of high level measurements.

## Discussion

Maternal antiretroviral therapy is an effective intervention to prevent breast milk HIV-1 transmission. In this study we evaluated breast milk at frequent intervals 2–3 times weekly for cell-free and cell-associated virus. Breast milk HIV-1 RNA was detected in more than half of the women receiving HAART, and women with breast milk HIV-1 RNA detection were more likely to be primiparous. The detection of breast milk HIV-1 RNA was strongly associated with concurrent plasma RNA detection and quantity. Consistent with previous reports [4], [6], we observed no association between breast milk HIV-1 RNA and breast milk HIV-1 DNA. Since breast milk HIV-1 RNA loads were closely related to plasma viral replication we infer that starting HAART earlier would result in better systemic suppression by delivery, and subsequently enable more complete suppression of HIV-1 RNA in breast milk.

Though the majority (87%) of breast milk specimens in this study had undetectable levels of HIV-1 RNA, 56% of women receiving HAART for PMTCT had at least one episode of detection. Most women had transient detection of breast milk HIV-1 RNA but two (8%) had detection at more than half their visits. In some clinical trials of breastfeeding women receiving PMTCT HAART, transmission rates have been as low as <2% [15], [16], [17], [21], [22]. Both cell-associated and cell-free virus could have significance for breast milk HIV-1 transmission [1], [2], [3]. The high efficacy of HAART in reducing transmissions supports cell-free RNA as the major contributor to transmission; the residual transmission risk observed in these studies despite HAART could be explained by episodic emergence of free virions in the milk (HIV-1 RNA), or persistence of the viral reservoir in breast milk cells (HIV-1 DNA).

In order to better understand mechanisms underlying episodic breast milk HIV-1 RNA detection during HAART, we studied relationships between viral replication in the blood and breast milk compartments. We found a strong correlation between breast milk HIV-1 RNA detection and both the detection and level of HIV-1 plasma RNA. In contrast, we did not find any association between breast milk HIV-1 RNA detection and breast milk HIV-1 DNA level. These data are consistent with findings from Shapiro and colleagues who reported no correlation between breast milk cellular DNA level and whole milk HIV-1 RNA levels at 2–5 months postpartum in women receiving ZDV plus single-dose NVP [6].

Our finding that breast milk viral levels are more closely related to plasma virus levels is also consistent with two recent phylogenetic studies conducted in Malawi [23] and Zambia [24], both of which found evidence for substantial mixing of plasma and breast milk viral RNA sequences, and ongoing seeding of the milk by viruses from the blood. The temporal correlation of intermittent viremia in these two compartments suggests that virus likely moves rapidly between these tissues, with viral breakthrough in the blood being rapidly reflected by breakthrough in the breast milk. Our data suggests that during the early weeks of HAART initiated for PMTCT, episodic breast milk viremia is governed more by movement of virus from the plasma than by local replication of virus in breast milk cells.

The episodic detection of plasma viremia during suppressive HAART has been interpreted as either viral breakthrough or false positive assays [8], [9], [10], [11]. The highly significant and large association between plasma and breast milk HIV-1 RNA detection we observed suggests that episodic detection of breast milk RNA in women starting HAART for PMTCT is not due to a random distribution of false-positive assays. Our sensitivity analysis further supports the validity of these measurements, since the association between HIV-1 RNA and plasma RNA was maintained when excluding the few higher-level measurements.

In this study, baseline immunosuppression or plasma HIV-1 RNA, time on HAART, response to HAART, or adherence did not differ significantly between women with and without breast milk HIV-1 RNA detection. Primiparity was associated with the detection of HIV-1 RNA in breast milk, which could imply breastfeeding technique, breast physiology, or mastitis might play a role in local HIV-1 reactivation. More study is needed to confirm this finding and determine the biologic mechanism explaining this association.

In this cohort, more than 70% of women had detectable low-level (median 2.7 log_10_ RNA copies/ml) plasma HIV-1 RNA at delivery. It is important to note that since women had been on HAART for only ∼5 weeks at delivery, the high frequency of women with plasma viremia does not indicate treatment failure. The 2010 WHO guidelines recommend initiation of HAART by 14 weeks gestation [25] and adults starting HAART are expected achieve viral suppression to <50 copies/ml within 16–24 weeks of therapy (MMWR 51-RR07). The degree of viral suppression in this cohort is consistent with data from other pregnancy cohorts, which report 42–96% of women starting HAART earlier in the third trimester achieving suppression by delivery [17], [21], [26], [27], [28]. The lower rate of suppression in our study may reflect differences in adherence of women starting later in pregnancy, the restricted CD4 eligibility criteria, and the relatively high viral load of women in our cohort.

Strengths of our study include frequent sampling of breast milk in the first month postpartum and the concurrent measurement of cell-free and cell-associated virus. Limitations include imprecision in measuring adherence; the time interval was different between breast milk collection and adherence assessment, which makes it difficult to determine whether temporal variations in adherence explain episodes of breast milk HIV-1 RNA detection. Breast milk HIV DNA measurements were not available for all women, because many specimens collected after 1 month postpartum did not contain adequate cell numbers for cryopreservation. As previously reported, there were no breast milk HIV-1 transmissions in the cohort, so we were not able to evaluate the relevance of breast milk HIV-1 RNA on transmission [7]. Selection of women with a narrow range of CD4 counts, and initiation of HAART relatively late in gestation also limits generalizability of these findings. Most women in the study initiated HAART during their third trimester; since there was little variability in time on HAART, we were not powered to examine the effect of HAART duration on HIV-1 RNA detection.

In summary, breast milk HIV-1 RNA was commonly detected in Kenyan women who had started HAART late in pregnancy and was associated with systemic HIV-1 replication, but not local breast milk HIV-1 DNA. These data suggest that plasma viral suppression may be a marker for HIV-1 transmission risk during pregnancy, and that earlier HAART may help reduce early breastfeeding transmission risk. The lack of association between HAART and breast milk HIV-1 DNA levels challenges the role of cell-associated virus in breast milk transmission. Whether episodic breast milk HIV-1 RNA or cell-associated DNA are responsible for the very low rates of transmission in women on HAART for PMTCT remains an open question requiring further study.

## Supporting Information

Figure S1 **Maternal virologic response to HAART.** Individual trajectories for plasma viral load during HAART are shown by gray lines. Black lines show linear models fit to plasma viral loads in antenatal (solid line) and postpartum (dashed line) visits.
(EPS)
